# Pneumonia, Acute Respiratory Distress Syndrome, and Early Immune-Modulator Therapy

**DOI:** 10.3390/ijms18020388

**Published:** 2017-02-11

**Authors:** Kyung-Yil Lee

**Affiliations:** 1 Department of Pediatrics, College of Medicine, The Catholic University of Korea, Seoul 06591, Korea; leekyungyil@catholic.ac.kr; Tel.: +82-42-220-9540; Fax: +82-42-221-2925; 2 Department of Pediatrics, Daejeon St. Mary’s Hospital, College of Medicine, The Catholic University of Korea, Daejeon 34943, Korea

**Keywords:** pneumonia, acute respiratory distress syndrome, pathogenesis, protein-homeostasis-system, corticosteroid, intravenous immunoglobulin

## Abstract

Acute respiratory distress syndrome (ARDS) is caused by infectious insults, such as pneumonia from various pathogens or related to other noninfectious events. Clinical and histopathologic characteristics are similar across severely affected patients, suggesting that a common mode of immune reaction may be involved in the immunopathogenesis of ARDS. There may be etiologic substances that have an affinity for respiratory cells and induce lung cell injury in cases of ARDS. These substances originate not only from pathogens, but also from injured host cells. At the molecular level, these substances have various sizes and biochemical characteristics, classifying them as protein substances and non-protein substances. Immune cells and immune proteins may recognize and act on these substances, including pathogenic proteins and peptides, depending upon the size and biochemical properties of the substances (this theory is known as the protein-homeostasis-system hypothesis). The severity or chronicity of ARDS depends on the amount of etiologic substances with corresponding immune reactions, the duration of the appearance of specific immune cells, or the repertoire of specific immune cells that control the substances. Therefore, treatment with early systemic immune modulators (corticosteroids and/or intravenous immunoglobulin) as soon as possible may reduce aberrant immune responses in the potential stage of ARDS.

## 1. Introduction

Acute respiratory distress syndrome (ARDS) or severe acute lung injury is a critical syndrome caused by heterogeneous etiologies, and is characterized by acute progression of respiratory symptoms and signs, bilateral diffuse infiltrates on chest imaging, and severe hypoxemia [1]. The severity of ARDS is associated with poor prognosis and higher mortality, and, by the Berlin definition, diagnostic hypoxemia is defined as decreased arterial PaO_2_/FiO_2_ ratio with parameters of 201–300 mmHg for mild ARDS, 101–200 mmHg for moderate ARDS, and ≤100 mmHg for severe ARDS [2].

Lungs perform the critical function of supplying oxygen to every cell of the body, and consist of a combined structure of a basic architecture of terminal airways, termed terminal bronchioles. The terminal structures are composed of several respiratory cell types including respiratory epithelial cells, endothelial cells, other stromal cells, and alveolar macrophages, as well as other organ-specific cells that commonly occur in the terminal structures of each organ. Therefore, the pathogenesis of ARDS is most simply described as extensive acute injury of a specific kind of respiratory cell directly by various insults, including infectious agents and/or host immune responses, or secondarily by ischemic insults, such as pulmonary thromboembolism or near drowning. Infectious factors such as pneumonia with/without sepsis caused by a variety of pathogens, including pneumococci, influenza viruses, coronaviruses, and malaria can be the cause of ARDS [3,4,5,6,7,8,9,10,11,12,13,14,15,16,17,18,19,20,21,22,23]. Also, various non-infectious factors, such as aspiration of gastric contents, near drowning, blunt chest contusion, multiple injuries, inhalation burns, pancreatitis, and multiple blood transfusions are associated with ARDS [24,25,26,27,28,29,30,31,32,33,34,35].

Although pathogens themselves, including viruses and mycoplasmas, are believed to be responsible for lung cell injury, the precise mechanism of lung injury in pneumonia at the molecular level remains unknown. Currently, it is understood that fragments of pathogens, including toxins and pathogen-associated molecular patterns (PAMPs), as well as substances from injured host cells from infectious insults, such as damage (danger)-associated molecular patterns (DAMPs), a category that includes heat shock proteins, can induce immune reactions [36,37,38]. Substances from activated immune cells, such as excessive cytokines and proteolytic enzymes, are also involved in lung injury.

Because the same kinds of immune cells and immune proteins, including immunoglobulins and complements, are observed in the pathologic lesions of pneumonia, ARDS, and other organ-specific pathologic lesions, it may be a reasonable assumption that immune systems function in the same way to protect against tissue cell injuries caused by various insults and to control toxic substances across organs. Also, it is proposed that all biological phenomena in organisms are controlled by a network termed the protein-homeostasis-system (PHS), and the immune system is one aspect of the PHS of organisms. In the PHS hypothesis, every human disease has etiologic substances and each immune cell in a host recognizes and acts against substances that are toxic to the target cells of the host, depending upon the size and biochemical properties of the substances, including pathogenic proteins (PPs) and pathogenic peptides [39,40,41].

This article discusses unresolved issues regarding the pathogenesis of pneumonia and ARDS. In addition, the article proposes a unified immunopathogenesis of ARDS, and briefly discusses early immune-modulator therapy for ARDS under the PHS of the host.

## 2. Etiology of Acute Respiratory Distress Syndrome (ARDS)

Various pathogens cause pneumonia, and occasionally pneumonia can progress to ARDS, multiple organ failure, and death (Table 1). The pathogens causing pneumonia may be regarded as the cause of ARDS, and the immunopathogenesis of ARDS may be the same as that of pneumonia. Thus, the extent of the area of lung injury may determine the clinical phenotypes of the lung injury.

The majority of patients infected with respiratory pathogens such as influenza viruses and *Mycoplasma pneumoniae* (*M. pneumoniae*) are asymptomatic or have mild symptoms, and only a proportion of patients manifest symptoms of systemic illness such as fever, fatigue, myalgia, and cough. According to the findings of a previous study, among patients with systemic symptoms, a small proportion had pneumonia. Among these pneumonia patients, only a smaller proportion of patients developed progressive pneumonia and ARDS [42]. Although risk factors such as being an infant, being elderly, comorbidity with other diseases, and immunodeficient states are associated with the development of ARDS in various respiratory infections, including influenza virus infection, previously healthy patients can also develop ARDS. For example, in the 1918 Spanish flu (H1N1 influenza virus) epidemic and past measles disasters in the South Pacific islands, populations of all ages were impacted. In these examples, mortality rates were very high, and paradoxically, mortality rates were highest in the healthiest age group (i.e., 20–40 years of age), which typically comprises individuals with the most active immune function [5,42,43]. These findings suggest that host factors (i.e., immune reactions against insults from pathogen infection) may determine the phenotype, severity, and prognosis of a disease.

In terms of ARDS, various non-infectious etiologic conditions, such as blunt chest contusion, multiple injuries, aspiration of gastric contents, inhalation burns, pancreatitis, near drowning, and multiple blood transfusions have been reported (Table 1). Although the pathogenesis or etiologic agents of ARDS in these conditions remain unknown, there should be etiologic substances that induce inflammation with immune cells and induce lung cell injury.

## 3. Histopathologic Findings and Unresolved Issues in ARDS

The pathologic findings of pneumonia and ARDS may vary according to etiologic agents and the stage of the disease. However, typical pathologies of advanced ARDS patients and animals show similar findings, including diffuse alveolar damage, hyaline membrane formation along alveolar walls, and extensive immune cell infiltration such as neutrophils and T cells in lung parenchyma and around damaged alveoli [44,45]. The state of distribution of immune cells and immune proteins in pneumonia lesions may inform the etiology and severity of lung disease. For example, neutrophils and phagocytic monocytes may be the predominant cells found in early-phase lesions of pneumococcal pneumonia, whereas small lymphocytes are the predominant immune cells in early lesions of viral or mycoplasma pneumonia [39,46], and eosinophils are the predominant cells in eosinophilic pulmonary diseases [47]. In addition, precipitated immunoglobulins in pathologic lesions also vary according to disease entities. For example, the classes and location of precipitated immunoglobulins (IgG, IgA, and IgM) vary according to renal disease entities [48]. Thus, the migration of immune cells and immune proteins into pathological lesions of ARDS is thought to be a necessity of immune reaction, as opposed to a non-specific or bystander reaction from chemokine stimulation in local lesions.

It has been believed that respiratory pathogens themselves, especially small pathogens such as respiratory viruses and mycoplasmas, induce inflammation in pathologic lesions, and spread to lower respiratory cells along the respiratory tracts. However, intact viruses are not found in a large proportion of patients with fatal outcomes or experimental animals with extensive pathologic lesions of ARDS caused by pathogens including pandemic influenza viruses [39,49]. Thus, the pathogens themselves may be too large to act as direct toxins to the host cells at the molecular biological level in vivo. Characteristic signs and symptoms of systemic infectious diseases, such as fever onset and various tissue injuries, may be initiated by an abrupt release of toxic substances from initial infection sites to systemic circulation. Nearly all infectious diseases, including pneumonia, may have a primary infection site (the focus) where pathogens replicate and where toxic substances are produced and released into nearby local lesions or systemic circulation. Extracellular bacterial infections (such as pneumococcal pneumonia and scarlet fever), intracellular bacterial infections (such as typhoid fever and legionella), and intracellular viral infections and small bacterial infections (such as mycoplasma pneumonia) may have initial foci for the replication of pathogens. In bacterial infections, including pneumococcal infection as an extracellular pathogen, the focus of a replication site may produce a lot of substances. These substances include the bacteria, fragments of bacterial components, such as polysaccharide capsules and other PAMPs, bacterial exotoxins, such as pneumolysin and bacteriosin, other materials from injured cells, including heat shock proteins and other DAMPs, and proteins from activated immune cells, such as proinflammatory cytokines and proteolytic enzymes [3,4,41]. Small pathogens such as viruses, mycoplasmas, rickettsiae, and chlamydias, and larger pathogens, such as parasites, will invade a host and establish a focus, which contains multiplied pathogens, substances or fragments of the pathogen-origin, materials from damaged cells, and other toxic substances to the host cells as well as in bacterial infections. During multiplication of the pathogens, the types of toxic substances, including virulence factors, may be dependent on the genomic sizes of the pathogens. In infectious diseases, the terms bacteremia and viremia could be interpreted as the state of systemic spread of pathogens and these related substances. Accordingly, a prognosis of ARDS in bacterial sepsis may be graver in comparison to that in other conditions [50].

Although pathogens are detected around a replication site on the upper respiratory tract in respiratory diseases, including influenza viruses, corona viruses, mycoplasmas, and legionellas, specific IgM and IgG antibodies to pathogens are not detected for at least three to four days after the onset of fever and clinical symptoms. This finding suggests that specific antibodies against the substances from pathogens are not produced in the initial replication stage, that is, during the incubation period in the host. Instead, systemic immune responses against pathogens and fragments of the pathogens may begin after the invasion of these substances into the systemic circulation of the host. After antibody formation, pathogens disappear rapidly in local lesions and systemic spread lesions, suggesting the important role of adaptive immune response in pathogen removal.

Animals with depressed T cell function or loss of T cell function such as nude mice show milder or few pneumonia lesions in comparison to immune-competent animals in mycoplasma or influenza virus infection models, although the duration of pathogen detection in the lungs of animals with compromised T cells is longer [51,52,53,54]. This finding suggests that lung injury is associated with T cell activation rather than with pathogens. During the clinical course of pneumonia, extrapulmonary manifestations such as skin rashes, myositis, meningoencephalopathy, and other organ involvement can occasionally occur. Some pathogens, such as mycoplasmas, exhibit no cytopathic effects on organ cells, with the exception of ciliated respiratory epithelial cells in vitro, and few intact pathogens are seen in pulmonary and extrapulmonary lesions [55]. Whether lower respiratory tract cells are damaged directly by a wide variety of small pathogens in vivo remains unclear. It is also unclear whether extrapulmonary cells have appropriate receptors for a variety of viruses to enter the target cells. There are critical organ-specific diseases that can seriously damage whole organs within a short period of time, including ARDS of the lungs. Examples of these critical organ-specific diseases are fulminating hepatitis (liver), acute myocarditis (heart), rapidly progressive glomerulonephritis (kidneys), acute necrotizing pancreatitis (pancreas), acute encephalopathy (including Reye syndrome) (brain), Waterhouse–Friderichsen syndrome (adrenal glands), toxic epidermolysis (skin), and acute hemorrhagic shock syndrome [56,57,58,59,60,61,62,63,64]. These organ-specific diseases are associated with more than one etiologic agent, including various pathogens. Generally, each organ-specific disease may have similar pathologic findings of extensive terminal organ cell damage with massive immune cell infiltration, as in ARDS. Also, direct detection of intact pathogens is difficult in extensive pathologic lesions, with the exception of occasional positive results on polymerase chain reaction. 

It is important to note that not only pathogen-derived substances, but also host cell-derived substances can be the cause of ARDS and other acute organ-specific diseases. There are several diseases that are associated with host cell damage. Damage inflicted upon the cells of one organ can affect the cells of other organs or the cells of the original organ. For example, sympathetic ophthalmia occurs after traumatic injury to one eyeball, and inflammation can occur within the ocular tissues of both eyeballs. If not treated promptly with corticosteroids or enucleation of the injured eye, blindness can occur in both eyes [65]. Rhabdomyolysis is an acute muscle cell injury caused by various factors such as infections with influenza or measles, physical injuries, and drugs as well as in ARDS [66,67]. Rhabdomyolysis can cause acute kidney injury, disseminated intravascular coagulation (DIC) of the blood, or heart arrhythmia. These complications may be due to systemically circulating extensive substances derived from the injured muscle cells, including myoglobin, potassium, and other toxic materials, to target cells [68]. Accordingly, this assumption may explain non-pathogen-induced ARDS, such as what occurs from blunt trauma, multiple tissue injuries, pancreatitis, severe burns, and other causes.

It is now accepted that the products of activated immune cells, including proteolytic proteins such as myeloperoxidases and other proteinases by neutrophils, major basic proteins and eosinophil cationic proteins by eosinophils, and proinflammatory cytokines such as TNF-α, can induce inflammation in various tissues, including lung tissues in ARDS [3,4,5,6,7,69,70,71]. In rapidly progressive organ-specific diseases, substances deriving from an initial focus may have an affinity for receptors on organ-specific cells. Additionally, toxic substances could be produced from organ-specific cells that are injured by an initial insult, as in the case of sympathetic ophthalmia. Because immune cells may activate and control target substances, communication across immune cells may be needed. During this process, excessive proinflammatory cytokines are produced, which may also be associated with rapid progression of certain organ specific diseases. The mechanism by which this cytokine imbalance induces host cell injury, however, remains to be elucidated.

To summarize, there are etiologic substances that induce inflammation in pathologic lesions of ARDS and other organ-specific diseases, and immune cells and immune proteins in these lesions may have a role in the recovery of the host from disease. Because all etiologic substances are controlled by immune cells and immune proteins, the role of each immune cell and immune protein, theoretically, may be the same in the pathologic lesions of ARDS caused by various etiologies.

## 4. Immunopathogenesis of Pneumonia and ARDS

The immune system of a host controls pathogens that have invaded the host, and thereby determines the prognosis of patients with any infectious diseases including pneumonia. The main role of the immune/repair system of the host is believed to be prevention of tissue cell injuries and repair of tissue cell damage from infectious insults, including various virulence factors. A variety of virulence factors in different infections have been investigated. In virus infections such as influenza or corona virus infection, the degree of affinity of viruses to lower respiratory tract cells is an important factor, while few secretory toxic substances have been reported, because of the small number of gene products (10 genes or more genes) associated with influenza or severe acute respiratory syndrome (SARS) [5,6,7]. In *M. pneumoniae* pneumonia, it is reported that cell membrane components, such as lipoproteins and secretory Community-Acquired Respiratory Distress Syndrome Toxin, may induce respiratory epithelial cell injury [46,72]. In pneumococcal infections, structural components of the bacteria, including capsule polysaccharides, bacterial DNA, lipotechoic acids, pneumococcal surface proteins, and choline-binding proteins, as well as secretory proteins, including pneumolysin and bacteriosin, have been proposed to be inducers of lung inflammation and lung cell injury [3,4]. How these diverse substances induce lung cell injury, however, requires further research studies.

Although the immune systems of mammals have been classified as innate (or natural) and adaptive (or specific) immune systems, both types of immune cells are found in nearly all pathologic lesions from infectious diseases, rheumatic diseases, cancers, transplantation rejection, and regeneration of tissues (keloids). Accordingly, it is believed that two types of immune system cells may work mutually against infectious or non-infectious insults, and that the function of both types of immune cells and immune proteins may be identical across a variety of pathologic lesions, as previously mentioned [41]. Both types of immune system cells communicate with each other through major histocompatibility complexes (MHCs) and protein-networks (cytokines) during events of external and internal insults. Any abnormalities in either type of immune cell or blockage of cross-talk proteins in immune reactions will result in delayed removal of pathogens in respiratory infections, including pneumococcal pneumonia and influenza virus infection [3,4,5,6,7].

Significant questions remain regarding what controls these substances and how the substances are controlled. Etiologic substances that induce lung cell injury may have various sizes, and can originate from external pathogens and/or from host cells, as previously discussed. The substances can be classified by biochemical characteristics as protein substances and non-protein substances. In addition, it is proposed that innate immune system cells, including natural antibodies, may control the non-protein substances, and that adaptive immune system cells may control the protein substances [41]. In innate immune system cells, neutrophils and phagocytic monocytes control larger substances, such as whole bacteria and viruses, as well as large pieces from destructed cells, such as apoptotic bodies or necrotic debris through phagocytosis. They work more effectively together with the products from other immune cells, such as antibodies and complements. For small non-protein substances, innate immune system cells have various receptors for fragments of pathogens, including PAMPs such as bacterial lipopolysaccharides (LPS), bacterial or viral DNAs and RNAs. In cases of virus infections, the receptors, termed pattern recognition receptors (PRRs), including Toll-like receptors (TLRs) and intracellular sensors (nuclear oligomerization domains and other examples) are bound with PAMPs such as viral DNAs or RNAs. This binding produces anti-pathogenic proteins, such as interferons and interferon-related proteins, proinflammatory cytokines, and other proteins [73,74]. Because both types of immune system cells may communicate with each other in all immunological events, the proteins produced by innate immune system cells may affect the function of adaptive immune system cells, including the expression of costimulatory receptors on adaptive immune cells and the differentiation of T cell subtypes [73]. Natural antibodies, as particular parts of the innate immune system, may also control non-protein substances, such as polysaccharides and non-protein substances from injured host cells [75]. Adaptive immune system cells, T cells and B cells, may control protein substances using T cell receptor (TCR) and B cell receptor (BCR) gene recombination. To briefly describe this process, B cells control medium-sized proteins via the production of antibodies, while T cells control small peptides that cannot induce antibodies via potential T cell receptor (TCR) gene-related immune reaction. For example, the PRRs of innate immune cells may work against small non-protein toxic substances from pathogens such as PAMPs, while the BCRs or TCRs of adaptive immune system cells work against protein toxic substances from the same pathogens. These receptor-antigen bindings do signal transduction and produce new proteins for controlling pathogens, such as anti-pathogen proteins or pathogen-specific antibodies [41].

Because the clinical course and pathologic findings of ARDS tend to be similar regardless of underlying disorders, the immunopathogenesis of ARDS can be explained as follows. Substances that are toxic to a kind of lung cells originate from an infectious focus or from damaged host cells in non-infectious conditions, and subsequently bind to the receptors of target respiratory cells. The affected respiratory cells signal to produce new proteins for communication with immune cells. Additionally, the process may damage the target cells and release the cell contents, including various-sized substances, the PPs and pathogenic peptides, and which recruit corresponding immune cells for the control of exposed substances. In the early stage of ARDS, innate immune system cells such as neutrophils, non-specific T cells, non-specific antibodies (B cells), other immune proteins, and possibly immune peptides, may comprise the first-line effectors for control of various-sized substances and may induce inflammation in pathologic lesions. However, non-specific T cells and non-specific antibodies are not as effective as specific effectors in their particular tasks. After the appearance of specific immune cells (T cells) and specific antibodies (B cells), the etiologic protein substances, including the PPs and pathogenic peptides, are effectively controlled and inflammation processes cease. Immune cells may communicate with each other through cytokine networks during this process, and the hyperactive immune phenomena against an extensive amount of substances, such as an imbalance of cytokines (i.e., a cytokine storm), may be responsible for further lung injury. If a host’s immune cell clones are unable to control the PPs and substances from injured lung cells, ARDS may progress to death in the acute stage or cause chronic inflammatory respiratory diseases due to the continuous activation of non-specific immune cells [41]. Therefore, it is possible that the virulence of pathogens, especially in viral infections, may lie in the ability of the pathogens to replicate with subsequent host cell injury following invasion, rather than the ability of the pathogens to produce toxins.

To summarize, the substances in ARDS (which are rapidly and massively produced within a short period of time from pathogen replication foci and/or from damaged cells) bind to target lung cells via systemic and/or local circulation systems. The various kinds of substances may have a similar mode of binding and signaling to lung tissue cells, and may be responsible for the recruitment and activation of corresponding immune cells and acute lung injury.

## 5. Treatment

Because the severity of pneumonia and ARDS may be dependent on the amount of substances that are toxic to respiratory cells, the first target of early treatment for ARDS is to reduce the toxic substances as soon as possible. Early antimicrobial therapy, such as the provision of antibiotics and antivirals, for pathogen-induced pneumonia is critical to reduce the number of pathogens and pathogen-originated substances, thereby inducing early recovery from the disease. Antibiotic treatment is recommended as soon as possible when bacterial infection is suspected. On the other hand, the use of antibiotics is not always successful in patients with community-acquired pneumonia (CAP). Some patients with bacterial pneumonia can experience complications such as lung abscess, empyema, pulmonary gangrene, and necrotizing pneumonia. Pneumonia has remained one of the most common causes of mortality in young children under five years of age in the developing world throughout the antibiotic era [76]. Furthermore, early treatment with antibiotics for young children with suspected pneumonia diagnosed by the clinical criteria of the World Health Organization has been shown not to reduce referral rates to hospitals or to prevent treatment failure, suggesting that most of these patients are affected by other non-bacterial respiratory pathogens [77]. Some pneumonia patients with CAP in developed countries, especially elderly patients with underlying diseases, experience treatment failure with a high mortality of 15%–20%, despite early application of antimicrobials [78,79]. Some pneumonia patients with septic conditions show transient deterioration of clinical symptoms following antibiotic treatment. This may be caused by a cytokine storm, characterized by extensive immune cell activation against large amounts of substances produced during the process of bacterial death [71]. Antibiotic treatment may induce rapid defervescence for patients with *M. pneumoniae* pneumonia, but some patients show progressive pneumonia despite early treatment with adequate antibiotics [80]. Necrotizing pneumonia is a unique type of lobar pneumonia caused by pneumococci and other pathogens [81]. Patients with necrotizing pneumonia show a protracted clinical course with prolonged fever, despite treatment with an adequate dose of antibiotics. Clinical course and computed tomography findings are relatively similar among patients affected with different pathogens, suggesting a common pathogenesis of the disease, such as ischemic lung injury caused by blood vessel occlusion from the insults of bacterial infection. Similar findings are observed in respiratory virus infections. In influenza virus infection, patients receiving early antiviral treatment such as oseltamivir may show more rapid defervescence than patients without early antiviral treatment. Some patients, however, are shown to be rapidly progressive to ARDS despite early antiviral treatment [82,83]. These findings suggest that antimicrobials may have limitations in some ARDS patients with infection-related conditions. 

Because abnormal immune reaction of the host against infectious insults, such as cytokine storm, is a suggested part of the immunopathogenesis of ARDS [3,4,5,6,7], early management of this type of immune disturbance may be critical in preventing the progression of the disease. Excessive substances from various insults react to a type of organ-specific tissue cells and induce corresponding excessive responses of immune cells, which may be responsible for damage to the same organ-specific cells, manifesting similar clinical and pathological findings. In order to reduce abnormal immune reactions, immune modulators, especially corticosteroids, have been used for pneumonia or ARDS. Although numerous studies, including studies regarding influenza pneumonia, have been conducted on corticosteroid effects in patients with severe pneumonia or ARDS, the results remain controversial [84,85,86,87]. The cause of this controversy, however, may be that the timing of therapy, the dose of initial steroids, schedules of treatment, and patient selection are different across existing studies. Recently, well-randomized case-control studies have reported that early corticosteroid treatment with antibiotics within 24–36 h after admission is helpful for reducing treatment failure and morbidity in adult patients with severe CAP [88,89]. Considering the immunopathogenesis of pneumonia and ARDS suggested in this article, earlier treatment (i.e., intervention as soon as possible) in fact stands to show better outcomes. We have also observed that early systemic immune modulators (corticosteroids and/or intravenous immunoglobulin (IVIG)) with antibiotics or antivirals may halt the progression of pneumonia and induce rapid recovery of pulmonary lesions in patients with *M. pneumoniae* or influenza virus infections [83,90,91,92]. In the 2009 influenza pandemic, we observed that extensive pneumonic consolidations that had developed rapidly within 48 h after fever onset resolved dramatically within 24 h after corticosteroids and/or IVIG treatment [83]. This finding suggests that there is a critical period for reversible pathologic states, which can be induced by early immune modulators. Acute bronchiolitis is a self-limiting lower respiratory tract infection in infancy, which is caused by various respiratory pathogens, including respiratory syncytial viruses, rhinoviruses, and *M. pneumoniae*. However, some severely affected patients show severe respiratory distress and complications, including respiratory failure with mechanical ventilation and subsequent bronchiolitis obliterans. Also, the effects of corticosteroid treatment for patients with acute bronchiolitis remain controversial despite a great deal of existing studies [93]. We have applied the same treatment modality for patients with severe acute bronchiolitis, as well as for severe *M. pneumoniae* and influenza pneumonia, which consist of early, short-term, high-dose and rapid tapering of corticosteroids. For severe bronchiolitis patients with respiratory distress in need of oxygen supply at the time of presentation or during hospitalization, we have used intravenous methylprednisolone (5–10 mg/kg/day, as initial dose), regardless of patient age and causal viruses. During the past decade at our institution, we experienced no patient who progressed to a state needing the intensive care unit (ICU) and mechanical ventilation or to respiratory complications among over 1200 patients (unpublished observation).

Lymphopenia may be characteristic of severe pneumonia patients infected with respiratory pathogens, including influenza viruses, corona viruses, the measles virus, and *M. pneumoniae* [94,95,96,97]. The severity of lymphopenia is correlated with the severity of lung injury [94,97]. The autopsy findings of severe ARDS patients and experimental animals infected with influenza viruses show lymphocyte depletion of whole lymphoid tissues [98]. This finding, together with lymphocyte predominance in early lung lesions, suggests that immune cells (including T cells) may control the substances from pathogens and/or injured host cells. It is possible that there is a limitation on the numerical capacity of the host immune system on mobilizing immune cells against these relentless substances to counter extensive lung cell injury in immune-competent patients. Patients with underlying diseases, malnutrition or immune-deficient states may have a limited repertoire of immune cells. Furthermore, severe pneumonia or ARDS from a viral infection tends to induce subsequent bacterial infections in patients, which adds to the workload of immune cells. However, prolonged high-dose corticosteroid therapy or immune-suppressants in advanced ARDS patients may suppress all working immune cells, including specific T cells and B cells that may control etiologic substances. Therefore, early management of conditions with ARDS potential may be crucial at the stage of hyperimmune reaction, possibly performed by non-specific adaptive immune cells. During any respiratory insult event, it is proposed that patients who have acute onset respiratory distress, such as dyspnea with or without wheezing, should be treated as soon as possible with an early and adequate dosage of systemic immune modulators (corticosteroids and/or IVIG). The rationale for this recommendation may be the same as the rationale behind the recommendations for early antibiotics and antivirals, since there may be a critical stage of lung cell injury due to hyperimmune reactions of the host [39,80,83]. The corticosteroid dose could be tapered rapidly for normally acting immune cells, especially for specific immune cells, which may appear within several days to a week from the time of insult.

Corticosteroids have multi-potent immune-modulatory and anti-inflammatory modes of action on almost all human diseases, including infectious diseases, allergic diseases, malignances, and rheumatic diseases. Although the entire mode of action of corticosteroids is unknown, corticosteroids may act on hyperactive immune cells that are needed for disease control. In the case of hyperactivity, however, these immune cells may overproduce immune substances such as proinflammatory cytokines. The immune cells affected by corticosteroids, especially non-specific immature T cells, B cells, and eosinophils, may be rapidly eliminated by apoptosis [99]. Intravenous immunoglobulin (IVIG) is an alternative immune-modulator, and indications for high-dose IVIG have been extended for immune-mediated diseases, including Kawasaki disease and other diseases [100]. It has been reported that IVIG shows beneficial effects on pulmonary lesions in influenza pneumonia and *M. pneumoniae* pneumonia [83,92]. Precise mechanisms of the immune-modulatory and anti-inflammatory effects of IVIG on immune-mediated diseases are also unknown, but IVIG may act on hyperimmune reactions of hosts via the binding to receptors of immune cells, etiologic substances including PPs, or other proteins that are involved in inflammatory pathways. Because corticosteroids (i.e., hydrocortisone) and IVIG (i.e., serum IgG) can be regarded as host-origin immune controllers in vivo, it is possible that a host immune system cannot produce them in adequate doses within the short duration of exposure to acute extensive substances from infectious insults. Thus, for patients with ARDS or other acute whole organ-specific diseases with lymphopenia, early systemic immune-modulator treatment before the occurrence of diffuse organ-specific cell injury may be critical, especially in previously healthy immune-competent patients. It is possible that an early and adequate dose of immune modulators can mitigate rapid disease progression, and reduce morbidity, and possibly prevent irreversible total organ destruction.

Although eventual recovery from ARDS is dependent on the immune status of a patient, other aspects of supportive care, especially lung preventive ventilation therapy, are important. The protective lung ventilation strategies (low tidal volume or limited driving pressure strategy) are currently accepted as major ways to improve the mortality of ARDS, and the main purpose of protective ventilation is to minimize the lung cell injury and avoid the further release of inflammatory mediators from the mechanically injured lung cells [101,102]. Other therapeutic modalities, such as extracorporeal membrane oxygenation (ECMO), nutritional support, and other anti-inflammatory therapies, are also important during the delicate period in which immune cells are combating the insults from ARDS.

## 6. Conclusions

Pneumonia and ARDS occur in heterogeneous conditions, but the immunopathogenesis of ARDS may be similar in different conditions. The present study presents a unified immunopathogenesis of ARDS using the PHS hypothesis. This hypothesis provides compelling reasons to unify the immunopathogenesis of ARDS and gives a rationale for early treatment with systemic immune modulators for patients in the beginning stage of ARDS. The severity or chronicity of ARDS depends on the amount of etiologic substances, including PPs and pathogenic peptides, the duration of the appearance of specific immune cells, or the repertoire of specific immune cells that control the substances in the host. Therefore, early systemic immune-modulator (corticosteroids and/or IVIG) therapy, administered as soon as possible, can reduce initial aberrant immune responses elicited by non-specific immune cells. This treatment policy for severe pneumonia or early ARDS can be described as having the same rationale as early antibiotic and antiviral therapies, insofar as there is a critical early stage of immune-mediated lung injury, which can be reversed with prompt intervention.

## Figures and Tables

**Table 1:** Causes of pneumonia and acute respiratory distress syndrome.

Infectious *	Noninfectious **
Bacterial	Aspiration of gastric contents
*Streptococcus pneumoniae*	Near-drowning
Group B streptococci	Pulmonary contusion
Group A streptococci	Toxic inhalation injury
*Staphylococcus aureus*	Multiple transfusions Pancreatitis
*Hemophilus influenzae*	Burns
	Drug overdose
*Neisseria* species Mixed anaerobes *Enterococcus* species *Mycoplasma pneumonia Chlamydia pneumonia* Mycobacterial *Mycobacterium tuberculosis* *Mycobacterium avium* Viral	Multiple bone fractures Post cardio-pulmonary bypass Traumatic brain injury
Influenza A,B	
Parainfluenza types 1–3	
Respiratory syncytial virus	
Coronavirus	
Adenovirus	
Metapneumovirus	
Measles virus	
Varicella zoster virus	
Fungal	
*Aspergillus* species	
*Blastomyces* species	
*Cryptococcus* species	
Parasitic	
*Pneumocystis jiroveci*	
Malaria	

* References [3,4,5,6,7,8,9,10,11,12,13,14,15,16,17,18,19,20,21,22,23], ** References [24,25,26,27,28,29,30,31,32,33,34,35].

## Abbreviations

ARDS	Acute respiratory distress syndrome
IVIG	Intravenous immunoglobulin
*M. pneumoniae*	*Mycoplasma pneumoniae*
PAMPs	Pathogen-associated molecular patterns
DAMPs	Damage (or danger)-associated molecular patterns
PHS	Protein-homeostasis-system
PPs	Pathogenic proteins
CAP	Community-acquired pneumonia
BCR	B cell receptor
TCR	T cell receptor
PRRs	Pattern recognition receptors
